# Case Report: Identification of two novel *ALMS1* variants in a patient with a ciliopathy resembling Alström syndrome

**DOI:** 10.3389/fgene.2026.1821427

**Published:** 2026-06-19

**Authors:** Chun-Qiong Ran, Mei Yang, Lin Chen, Xun Liu

**Affiliations:** Department of Endocrine, Liangjiang Hospital of Chongqing Medical University, Chongqing, China

**Keywords:** Alström syndrome, case report, genetic counseling, genetic testing, precision diagnosis

## Abstract

**Background:**

Alström syndrome (AS) is a rare autosomal recessive ciliopathy caused by biallelic pathogenic variants in the *ALMS1* gene. The condition is characterized by a spectrum of clinical manifestations, including cone-rod dystrophy, sensorineural hearing loss, metabolic disturbances, and progressive multiorgan involvement. Ciliopathies share considerable clinical overlap, and some cases present with features that resemble AS without meeting all diagnostic criteria. This study aims to identify the pathogenic variants in a Chinese patient with a ciliopathy resembling AS.

**Case Presentation:**

We report a 27-year-old Chinese female with a body mass index (BMI) of 28.4 kg/m^2^. The patient initially presented with progressive hearing loss at age 2 years, followed by visual impairment beginning at age 8 years. At age 17 years, she developed progressive alopecia accompanied by scaly, patchy papules. By age 22 years, she was diagnosed with polycystic ovary syndrome (PCOS) and type 2 diabetes mellitus (T2DM). Laboratory investigations revealed dyslipidemia and hyperinsulinemia. Abdominal ultrasonography demonstrated hepatic steatosis and medullary sponge kidney, while pelvic ultrasound indicated polycystic ovarian morphology. Echocardiography revealed ventricular septal thickening. Whole-exome sequencing (WES) identified that our patient was a compound heterozygote for two novel variants in the *ALMS1* gene, comprising c.12114 + 1G>T and c.1856_1860dup (p.Ser621Leufs*23). Both variants were classified as likely pathogenic in accordance with the American College of Medical Genetics and Genomics and the Association for Molecular Pathology (ACMG/AMP) guidelines. Based on the clinical phenotype and molecular findings, a diagnosis of a ciliopathy resembling AS was made, although a definitive diagnosis of AS could not be confirmed due to incomplete ocular phenotyping. Disease management included metformin and pioglitazone for T2DM, continued use of hearing aids, and scheduled regular follow-up evaluations.

**Conclusion:**

This report describes two novel *ALMS1* variants, expanding the known mutational spectrum of the gene. Genetic testing plays a supportive role in diagnosis and is valuable for familial screening and counseling, although a definitive diagnosis of AS would require complete ophthalmic phenotyping and functional validation.

## 1 Introduction

Ciliopathies are a group of genetically and phenotypically overlapping disorders caused by dysfunction of primary cilia (Focşa et al., 2021; Gerth-Kahlert and Koller, 2018). They share a broad spectrum of clinical features, often including retinal dystrophy, obesity, hearing loss, diabetes mellitus, renal abnormalities, and cognitive impairment (Focşa et al., 2021; Gerth-Kahlert and Koller, 2018). The considerable phenotypic overlap among different ciliopathies frequently leads to diagnostic challenges, especially when the ocular phenotype is incompletely characterized.

Alström syndrome (AS; OMIM #203800) is a rare autosomal recessive disorder first described by the Swedish physician Carl Henry Alström in 1959 (Alström et al., 1959). Its estimated prevalence ranges from 1 to 9 per million individuals, with no sex predilection, although the incidence is markedly higher among the offspring of consanguineous unions (Hearn, 2019; Choudhury et al., 2021). AS is characterized by progressive multisystem involvement, including neurosensory impairments such as cone-rod dystrophy and sensorineural hearing loss, as well as metabolic and endocrine abnormalities—most notably type 2 diabetes mellitus (T2DM), obesity, and short stature (Choudhury et al., 2021; Marshall et al., 2011). Additional complications frequently involve hepatic, renal, and pulmonary dysfunction, along with dilated cardiomyopathy (Choudhury et al., 2021; Marshall et al., 2011). Although clinical evaluation remains the essential first step in diagnosis, the progressive nature of multiorgan dysfunction and the variable expressivity observed even among affected family members pose considerable diagnostic challenges. Currently, in conjunction with clinical findings, the identification of biallelic pathogenic variants in the *ALMS1* gene is a cornerstone of the diagnostic criteria for AS (Marshall et al., 2007a). This was firmly established in 2002, when Collin et al. (2002) first demonstrated that biallelic mutations in *ALMS1* are causative of the disorder.

The *ALMS1* gene, located on chromosome 2p13.1, spans 23 exons and encodes a 4,169-amino acid protein with a molecular weight of 461 kDa (Hearn et al., 2002). The ALMS1 protein is ubiquitously expressed and localizes to the centrosome and basal body of cilia, implicating it in a range of fundamental cellular processes, including ciliary function, intracellular trafficking, cell cycle regulation, differentiation, and metabolic homeostasis (Hearn, 2019; Hearn et al., 2005). Biallelic pathogenic variants—either homozygous or compound heterozygous—in the *ALMS1* gene are responsible for AS. Pathogenic variants in *ALMS1* are predominantly truncating mutations, notably nonsense and frameshift variants caused by single-nucleotide changes or small insertions/deletions (DeMaagd and Engber, 1989; Ozantürk et al., 2015). These mutations introduce a premature termination codon, consequently leading to the production of a truncated protein and triggering a nonsense-mediated decay (DeMaagd and Engber, 1989; Ozantürk et al., 2015). Genomic analyses have identified exons 8, 10, and 16 as major mutational hot spots (Marshall et al., 2007b; Hu et al., 2024). Beyond these common alterations, a limited number of non-canonical variants have been reported, including chromosomal translocation, insertions of AluYa5 repetitive elements, large deletions, and splice-site mutations (Hearn et al., 2002; Bond et al., 2005; Taşkesen et al., 2012; Marshall et al., 2015; Nikopoulos et al., 2016; Saadah et al., 2021).

In this report, we describe a 27-year-old female presenting with T2DM, polycystic ovary syndrome (PCOS) with clinical features of hyperandrogenism (e.g., alopecia), sensorineural hearing loss, vision impairment, and fatty liver disease. To establish a definitive diagnosis, we performed whole-exome sequencing (WES) and conducted familial segregation analysis via Sanger sequencing. Our investigation revealed compound heterozygosity for two novel *ALMS1* variants, suggesting a ciliopathy resembling AS. However, due to incomplete ocular phenotyping, a definitive diagnosis of AS cannot be confirmed. This case highlights the diagnostic challenges posed by overlapping ciliopathy phenotypes and the necessity of comprehensive ophthalmic assessment even when genetic findings are present.

## 2 Materials and methods

### 2.1 Patient and clinical evaluations

**FIGURE 1:**
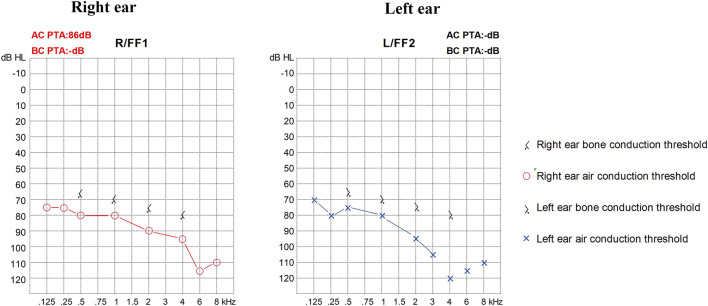
Audiometry of the patient. The pure tone audiogram indicates bilateral profound sensorineural hearing loss, particularly in the high-frequency range. AC, air conduction; BC, bone conduction; PTA, pure tone average.

We conducted comprehensive genetic and clinical assessments of the patient with AS and assessed the family history, focusing on consanguinity and preterm birth. The patient underwent a general clinical exam at the Department of Internal Medicine. The ophthalmological assessment included a comprehensive ophthalmic examination following standard clinical protocols, comprising non-contact tonometry (NCT), video nystagmography, color fundus photography, optical coherence tomography (OCT), static visual field testing, and visual evoked potential (VEP) assessment. Hearing tests included tympanometry and pure tone audiometry (Figure 1). Systemic evaluation involved serum testing for liver and renal function, electrolytes, lipid profile, blood glucose, C-peptide, and hormone level, in addition to abdominal ultrasonography, pelvic ultrasonography, and transthoracic echocardiography. This study was approved by the Medical Ethics Committee of Chongqing Liangjiang New Area People’s Hospital (Approval No. 2025-193). Written informed consent was obtained from the proband and both parents prior to their participation.

### 2.2 Gene detection and analysis

Genomic DNA was extracted from peripheral blood samples collected in EDTA tubes from the proband. WES was performed at the Chongqing Puroton Gene Medical Research Institute (Chongqing, 400712, China), and the resulting sequence reads were aligned to the human reference genome (GRCh37/hg19). The pathogenicity of identified variants was interpreted according to the American College of Medical Genetics and Genomics and the Association for Molecular Pathology (ACMG/AMP) guidelines (Richards et al., 2015), using the following classification categories: pathogenic, likely pathogenic, variant of uncertain significance, likely benign, and benign. Candidate variants identified by WES were validated by Sanger sequencing in the proband and her parents. Specific PCR amplification of *ALMS1* gene fragments was carried out using the following primer pairs: for the c.12114 + 1G>T variant, forward 5′-CCC​CTG​AGA​ACC​TGT​ATT-3′ and reverse 5′-ACA​GAT​GAT​GAG​AAA​CCC-3’; for the c.1856_1860dup variant, forward 5′-CAT​TTA​TCC​TTG​TCC​CTT-3′ and reverse 5′-CTG​TAG​GTA​TTC​CCG​TCT-3’. The frameshift variant was evaluated using online software MutationTaster (http://mutationtaster.org) for pathogenicity prediction. The potential impact of the splice-site variant on *ALMS1* RNA secondary structure was predicted using the RNAfold web server (http://rna.tbi.univie.ac.at/cgi-bin/RNAWebSuite/RNAfold.cgi), which uses a loop-based energy model and dynamic programming algorithm to predict secondary structures of linear or circular single-stranded RNA.

## 3 Results

### 3.1 Clinical manifestation

#### 3.1.1 Ophthalmic manifestations

**FIGURE 2:**
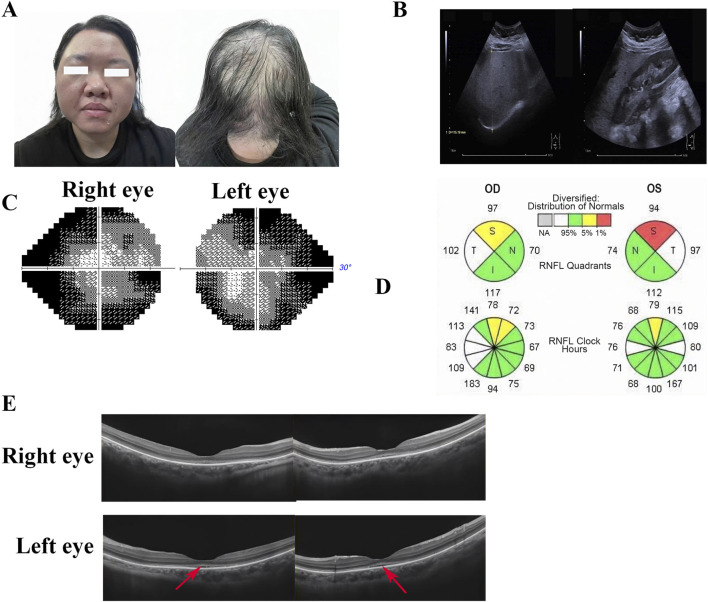
Clinical and imaging findings of the patient. **(A)** The patient presented with a round face and sparse hair. **(B)** Abdominal ultrasonography revealed hepatic steatosis and a medullary sponge kidney. **(C)** Perimetry demonstrated bilateral visual field defects. **(D,E)** Optical coherence tomography of the left eye revealed a shallow detachment between the neurosensory retina (photoreceptors) and the retinal pigment epithelium, with associated focal RNFL thinning.

The patient was a 27-year-old Chinese female. She first experienced visual impairment at age 8 years, which was initially attributed to bilateral congenital amblyopia. Ocular examination showed best-corrected visual acuity (BCVA) of 0.1 (20/200) in both eyes. Cycloplegic refraction revealed no myopia, no hyperopia, and no astigmatism. Intraocular pressure was bilaterally elevated (OD: 21.5 mmHg; OS: 24.7 mmHg). No nystagmus was observed clinically or on video nystagmography. OCT of the left eye (OS) showed a shallow detachment between the neurosensory retina (photoreceptor layer) and the retinal pigment epithelium (RPE), along with focal retinal nerve fiber layer (RNFL) thinning (Figure 2; Supplementary Figure S1). Perimetry identified bilateral constricted (tubular) visual fields, characterized by peripheral visual field loss with central sparing (Supplementary Figures S2, S3). VEP testing demonstrated bilaterally prolonged P100 latency, indicating abnormal electrical activity of the bilateral visual pathways (Supplementary Figure S4; Supplementary Table S1). Full-field electroretinography (ffERG) and fundus autofluorescence (FAF) imaging were not performed (patient declined), which precludes definitive characterization of the retinal dystrophy subtype and represents a limitation of this study.

#### 3.1.2 Systemic manifestations

The patient was born at full-term to a non-consanguineous family, and her first-degree relatives (parents and sister) were healthy with no known genetic disorders. She had a long-standing history of obesity dating back to childhood, with a body mass index (BMI) of 28.4 kg/m^2^ at admission. Bilateral hearing loss began at age 2 years, progressed over time, and was formally diagnosed as bilateral sensorineural hearing loss at age 17 years, necessitating hearing aids. At age 17 years, she developed scaly, patchy papules on the scalp, face, trunk, and limbs, accompanied by progressive alopecia, managed with topical glucocorticoids. By age 22 years, she was diagnosed with PCOS, manifesting as irregular menstrual cycles, and concurrently with T2DM.

Abdominal ultrasonography revealed hepatic steatosis and findings suggestive of medullary sponge kidney (Figure 2). Echocardiography showed thickening of the ventricular septum (maximum thickness: 12 mm), with normal left ventricular ejection fraction. Pelvic ultrasound indicated bilateral ovarian enlargement (left: 4.2 × 1.7 cm; right: 5.7 × 4.1 cm). The left ovary contained at least 12 follicles per cross-section, each <0.5 cm in diameter. The right ovary showed 7–8 follicles measuring 2–10 mm. These findings are consistent with a polycystic ovarian morphology. Routine laboratory tests were performed, and the results are summarized in Table 1.

**TABLE 1:** Laboratory findings of the patient.

Parameter	Value	Normal range
Glycemic parameters		
Fasting plasma glucose (mmol/L)	7.63	3.9-6.1
Fasting insulin (μU/mL)	30.14	3-25
C‑peptid (ng/mL)	4.54	0.81-3.85
HbA1c (%)	6.6	4-6.2
Blood lipid		
Total cholesterol (mmol/L)	4.53	2.80-5.17
Triglyceride (mmol/L)	1.49	0.01-1.7
HDL-cholesterol (mmol/L)	0.76	1.29-1.55
LDL-cholesterol (mmol/L)	3.6	0-3.37
Autoantibodies in diabetes		
GADA	Neg.	Neg.
ICA	Neg.	Neg.
IAA	Neg.	Neg.
IA-2A	Neg.	Neg.
Hepatic function		
ALT (U/L)	31	7-40
AST (U/L)	25	13-35
Renal function		
Creatinine (μmol/L)	68.3	41.0-73.0
Cardiac biomarkers		
CK (U/L)	46	40-200
LDH (U/L)	72.83	17-96
BNP (pg/mL)	29	0-100
Thyroid related indicators		
FT3 (pg/mL)	2.44	2.3-4.2
FT4 (ng/dL)	1.11	0.89-1.76
TSH (μIU/mL)	1.95	0.55-4.78
Sex hormones		
FSH (mIU/mL)	4.46	1.5-9.1
LH (mIU/mL)	5.99	1.9-12.5
Progesterone (ng/mL)	4.46	3.34-25.56
Testosterone (ng/dL)	27.90	9.01-47.94
Estradiol (pg/mL)	245.05	63.9-356.7
Prolactin (ng/mL)	7.89	2.8-29.2
DHEA‑S (ng/mL)	1477	180-4000
Adrenal function parameters		
Cortisol (nmol/L)	284.26	145.4-619.1
ACTH (pg/mL)	15.40	5.00-60.00

HbAlc, glycated hemoglobin; Neg., negative; IAA, insulin antibody; ICA, islet cell antibody; IA-2A, protein tyrosine phosphatase antibody; GADA, glutamic acid decarboxylase antibody; ALT, alanine aminotransferase; AST, aspartate aminotransferase; CK, creatine kinase; LDH, lactate dehydrogenase; BNP, B-type natriuretic peptide; FT3, free triiodothyronine; FT4, free thyroxine; TSH, thyroid-stimulating hormone; FSH, follicle-stimulating hormone; LH, luteinizing hormone; DHEA-S, dehydroepiandrosterone sulfate; ACTH, adrenocorticotropic hormone.

Following the diagnosis of a ciliopathy resembling AS, management included metformin and pioglitazone for T2DM, continued use of hearing aids, and scheduled regular follow-up evaluations.

### 3.2 Genetic findings

**FIGURE 3:**
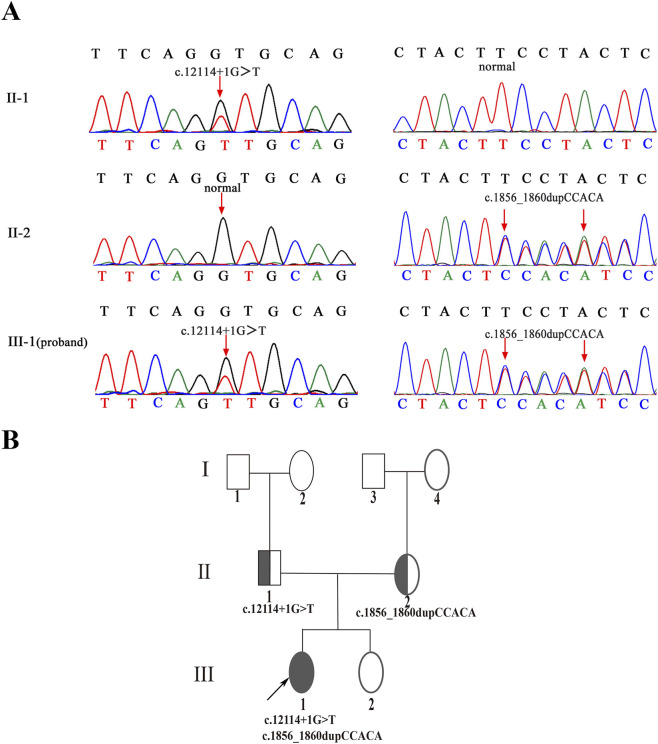
Genetic characteristics and pedigree of the family. **(A)** Sequencing analysis revealed that the proband (III-1) is a compound heterozygote for two novel variants: c.12114 + 1G>T and c.1856_1860dup (p.Ser621Leufs*23). **(B)** Pedigree of the family. Squares represent males and circles represent females. The arrow indicates the proband.

**FIGURE 4:**
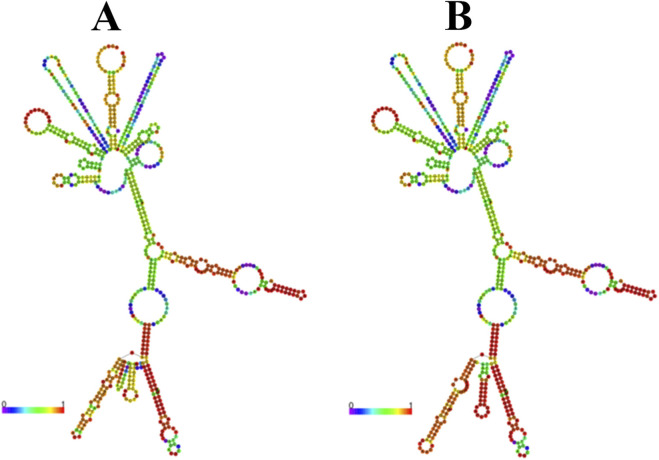
RNA secondary structure prediction. The *ALMS1* RNA secondary structures of native **(A)** and variant **(B)** types were predicted by RNAfold and revealed that c.12114 + 1G>T caused a marked change in the RNA structure of *ALMS1*. The bars at the bottom of the figure indicate base pairing probability.

We conducted a comprehensive genetic assessment of the patient with AS and her parents. Genetic testing identified the patient as a compound heterozygote for two novel variants in the *ALMS1* gene: c.12114 + 1G>T (canonical splice donor) and c.1856_1860dup (p.Ser621Leufs*23) (Figure 3). Segregation analysis by Sanger sequencing confirmed that the c.12114 + 1G>T variant, located in exon 19, was inherited from the father (II-1), while the c.1856_1860dup variant in exon 8 was inherited from the mother (II-2), establishing that the two variants are located in trans. Neither variant has been previously reported in the literature, and both were absent from public population databases, including the 1000 Genomes Project, ExAC, and gnomAD. However, the *ALMS1* c.12114 + 1G>T variant was documented in ClinVar (https://www.ncbi.nlm.nih.gov/clinvar/) with a classification of likely pathogenic. RNA secondary structure predictions using RNAfold (http://rna.tbi.univie.ac.at/cgi-bin/RNAWebSuite/RNAfold.cgi) indicated that the c.12114 + 1G>T variant leads to a significant alteration in RNA structure of *ALMS1* in both the patient and her father (Figure 4). MutationTaster predicted that the c.1856_1860dup variant is disease-causing (probability = 1.0). According to ACMG/AMP 2015 guidelines, the c.12114 + 1G>T variant meets criteria PVS1 (canonical splice variant expected to cause loss of function), PM2 (absent from population databases), and PM3 (in *trans* with another loss-of-function variant). The c.1856_1860dup variant also meets PVS1 (frameshift leading to premature termination), PM2, and PM3. However, due to the incomplete ocular phenotyping (lack of ERG and FAF), we conservatively classify both variants as likely pathogenic (Class 4). Functional studies and complete ophthalmic testing would be required to upgrade to pathogenic (Class 5).

## 4 Discussion

AS is a rare autosomal recessive ciliopathy caused by biallelic loss-of-function variants in *ALMS1*. The encoded protein localizes to the centrosome and the base of primary cilia, where it plays essential roles in maintaining ciliary structure and function, mediating intracellular transport, ciliary signaling, and cell differentiation (Joy et al., 2007; Marshall et al., 2007b). *ALMS1* is widely expressed in the central nervous system, retinal photoreceptors, endocrine organs, heart, liver, kidney, and inner ear, accounting for the multisystemic and highly variable clinical manifestations of AS (Choudhury et al., 2021; Tahani et al., 2020).

### 4.1 Clinical spectrum and comparison with reported splice-site variants

Retinal dystrophy represents a core feature of AS, typically presenting with photophobia and nystagmus and progressing to complete blindness by the second decade of life (Tahani et al., 2020; Jecan-Toader et al., 2024). This is frequently accompanied by bilateral sensorineural hearing loss, which develops in approximately 70% of patients before the age of 10 (Tahani et al., 2020; Jecan-Toader et al., 2024). Endocrine and metabolic disturbances are common, including early-onset obesity, insulin resistance, and T2DM. The underlying metabolic dysregulation is linked to enhanced insulin-mediated GLUT4 translocation, a process facilitated by *ALMS1* mutations through interactions with the actin cytoskeleton that promote cellular glucose uptake (Choudhury et al., 2021). Other frequent endocrine complications comprise hypertriglyceridemia and, in female patients, hyperandrogenism (Han et al., 2018). The diagnosis of AS, as per established guidelines, is based on this constellation of symptoms that worsen over time, supplemented by genetic confirmation. Identifying biallelic pathogenic variants in the *ALMS1* gene is central to definitive diagnosis. In the present case, genetic analysis confirmed that both the proband and her parents carried the *ALMS1* mutation. Consequently, the patient’s systemic phenotype is suggestive of a ciliopathy, although the absence of ffERG/FAF prevents complete confirmation of the retinal phenotype.

As shown in Table 2, the clinical phenotypes of previously reported *ALMS1* splice-site variants are summarized alongside the novel variant identified in our patient (c.12114 + 1G>T). Comparing our patient with these previously reported cases, we note that obesity, hearing loss, and diabetes are common but not universal. Dilated cardiomyopathy was absent in our patient, although echocardiography showed isolated septal thickening. Renal manifestations varied from microalbuminuria to medullary sponge kidney. Eye findings across all cases consistently included photophobia, reduced vision, and retinal degeneration, but the severity and specific features (e.g., nystagmus and optic atrophy) differed.

**TABLE 2:** Phenotypic spectrum associated with *ALMS1* splice-site mutations.

Clinical manifestation	Variant						c.7677 + 1G>T (Shi et al., 2023)	c.12114 + 1G>T
	c.11550 + 3A>T (Weiss et al., 2019)	c.11666–2A>G (Kılınç et al., 2018)	c.11876–3T>G (Sanyoura et al., 2014)	c.11876–2A>T (Aldahmesh et al., 2009)	c.11873–2A>T (Saadah et al., 2021)	c.9542G>A (Shi et al., 2023)		
Obesity	+	+	-	+	+	-	-	+
Dilated cardiomyopathy	+	+	-	-	+	-	-	-
Hearing loss	+	-	+	-	+	+	-	+
Diabetes	​	-	+	-	+	+	-	+
Short stature	+	-	-	+	+	​	-	+
Renal dysfunction	​	Microalbuminuria	+	-	-	+	-	-
Acanthosis nigricans	+	+	-	+	+	+	-	-
Hormone level	Hypothyroidism	-	Hypogonadotropic hypogonadism	-	Hypothyroidism, hypogonadotropic hypogonadism	-	​	Hyperandrogenism
Eyes condition	Nystagmus, vision loss	Photophobia, retinal dystrophy, vision loss	Early-onset retinopathy with optic atrophy, nystagmus Blindness	Nystagmus, retinal dystrophy, vision loss	Photophobia, nystagmus, retinal dystrophy, vision loss	Reduced vision, cone-rod dystrophy	Nystagmus, photophobia, reduced vision, squint	Photophobia, degenerative retinal changes Reduced vision

### 4.2 Genetic findings and variant classification

Currently, five major categories of *ALMS1* pathogenic variants have been documented, predominantly comprising frameshift, nonsense, and missense mutations (Cheng et al., 2022). Notably, frameshift and nonsense variants constitute the most frequent categories, and almost half of all variants occur in exon 8—a finding that may be explained by the unusually large size of this exon. In the present case, we identified two variants: c.1856_1860dup in exon 8 and c.12114 + 1G>T in exon 19. Segregation analysis confirmed that the two variants are in *trans*. The c.1856_1860dup variant is a frameshift insertion that leads to a premature termination codon 23 amino acids downstream (p.Ser621Leufs*23). This is predicted to result in nonsense-mediated mRNA decay or production of a truncated, non-functional ALMS1 protein. MutationTaster predicted this variant to be disease-causing with a probability of 1.0. Although it is novel, its loss-of-function nature and absence from population databases support likely pathogenicity. The latter is a splice-site mutation consistent with previously reported variants (Saadah et al., 2021). Based on its autosomal recessive inheritance pattern and supporting evidence from *in silico* analysis, we propose that this variant is likely disease-causative. Given the lack of experimental functional data, we performed a predictive analysis of the c.12114 + 1G>T variant using RNAfold software. This *in silico* analysis predicted that the variant alters the secondary structure and folding dynamics of *ALMS1* mRNA. The change in the secondary structure of the RNA resulting from the splice-site variant may further alter its function through two possible mechanisms. First, loss of correct RNA folding would abolish or alter its functionally critical interactions with protein complexes such as the spliceosome, thereby disrupting mRNA splicing, RNA transport, or both (Magnus et al., 2019). Second, the misfolded RNA may conceal or alter miRNA binding sites, compromising normal post-transcriptional regulation and contributing to disease etiology (Soemedi et al., 2017; Anna and Monika, 2018).

### 4.3 Genotype–phenotype correlation

Recent studies have attempted to elucidate correlations between clinical features and pathogenic variants in the *ALMS1* gene. Marshall et al. (2007b) reported a significant association between variants in exon 8 and absent, mild, or delayed renal disease while also identifying a suggestive link between variants in exon 16 and diabetes, cardiomyopathy, retinal degeneration, and urological dysfunction. A meta-analysis involving 357 patients with AS further revealed that variants in exon 10 were associated with higher rates of liver disease (Bea-Mascato and Valverde, 2023). Nevertheless, the study concluded that the location of the pathogenic variant has minimal influence on overall disease manifestations (Bea-Mascato and Valverde, 2023). Similarly, Ozantürk et al. (2015) observed no direct relationship between mutation type and clinical phenotype, despite the progressive multisystem involvement consistently observed in AS patients. In this study, we performed a literature review focusing on pathogenic splicing variants in *ALMS1* and found no clear association between these variants and specific clinical outcomes. A previous study has highlighted that identical mutations can lead to widely varying clinical characteristics, age of onset, and disease severity, even within families (Bea-Mascato and Valverde, 2023). This may be attributed to the fact that phenotypic expression can be modulated by modifying factors, such as environmental influences and infectious exposures.

### 4.4 Management

There is currently no cure for AS, and no disease-modifying therapy has been proven to prevent or reverse existing organ damage. Consequently, early diagnosis and intervention are essential to mitigate multi-organ progression and improve long-term prognosis and quality of life. Beyond systemic disease management, addressing associated comorbidities is critical. For hearing impairment, cochlear implants can restore auditory function, enabling communicative, social, and academic outcomes comparable to those of peers (Gheller et al., 2020). In metabolic management, lifestyle modifications—including a low-calorie, low-fat diet and regular physical activity—support weight loss and can improve glycemic and lipid profiles (Geets et al., 2019). Pharmacologically, metformin and thiazolidinediones (TZDs) are utilized to treat insulin resistance and T2DM in AS patients (Tahani et al., 2020). When weight loss is clinically indicated, glucagon-like peptide-1 receptor agonists (GLP-1 RAs) represent a recommended therapeutic option (Tahani et al., 2020). Notably, recent evidence highlights the dual GIP/GLP-1 agonist tirzepatide as effective in reducing body weight, improving metabolic dysfunction-associated steatotic liver disease, and ameliorating insulin resistance in AS (Ferch et al., 2025). In the present case, the patient was prescribed metformin and pioglitazone for T2DM, advised to continue using hearing aids, and scheduled for regular follow-up assessments.

## 5 Conclusion

In this case, we identified two novel variants in *ALMS1* (c.12114 + 1G>T and c.1856_1860dup) in a patient presenting with a ciliopathy phenotype resembling AS. Due to incomplete ocular phenotyping (lack of ffERG and FAF) and the absence of functional validation, a definitive diagnosis of AS could not be established. Our findings expand the mutational spectrum of *ALMS1* and highlight the diagnostic challenges posed by overlapping ciliopathy phenotypes. Comprehensive ophthalmic evaluation, including ffERG and FAF, functional studies, and whole-genome sequencing are needed to clarify the role of these variants and to achieve a precise diagnosis. This case underscores the importance of cautious interpretation of genetic findings when the clinical phenotype is incomplete.

## Data Availability

The raw data supporting the conclusions of this article will be made available by the authors, without undue reservation.
