# Supplementary material for: Case Report: Identification of two novel ALMS1 variants in a patient with a ciliopathy resembling Alström syndrome
Source: Front Genet. 2026 Jun 19;17:1821427. doi: 10.3389/fgene.2026.1821427 (PMC13327652; doi:10.3389/fgene.2026.1821427)
Supplement: Supplementary file 1 [file DataSheet1.docx]

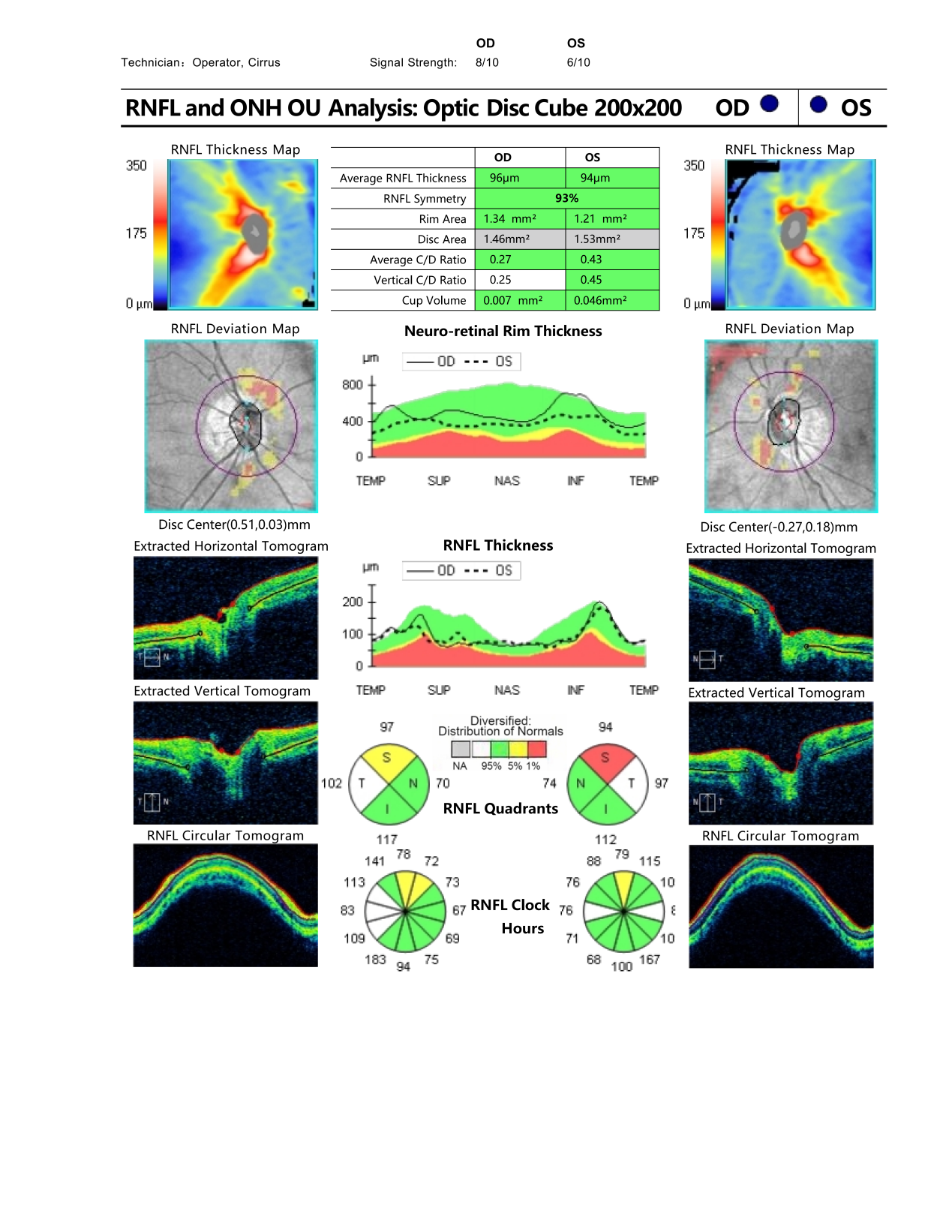


**Supplementary Figure. 1 **OCT-RNFL report of the patient.**** The report demonstrates mild thinning of the superior RNFL in both eyes. OCT, o**ptical coherence tomography; RNFL,retinal nerve fiber layer.**

**
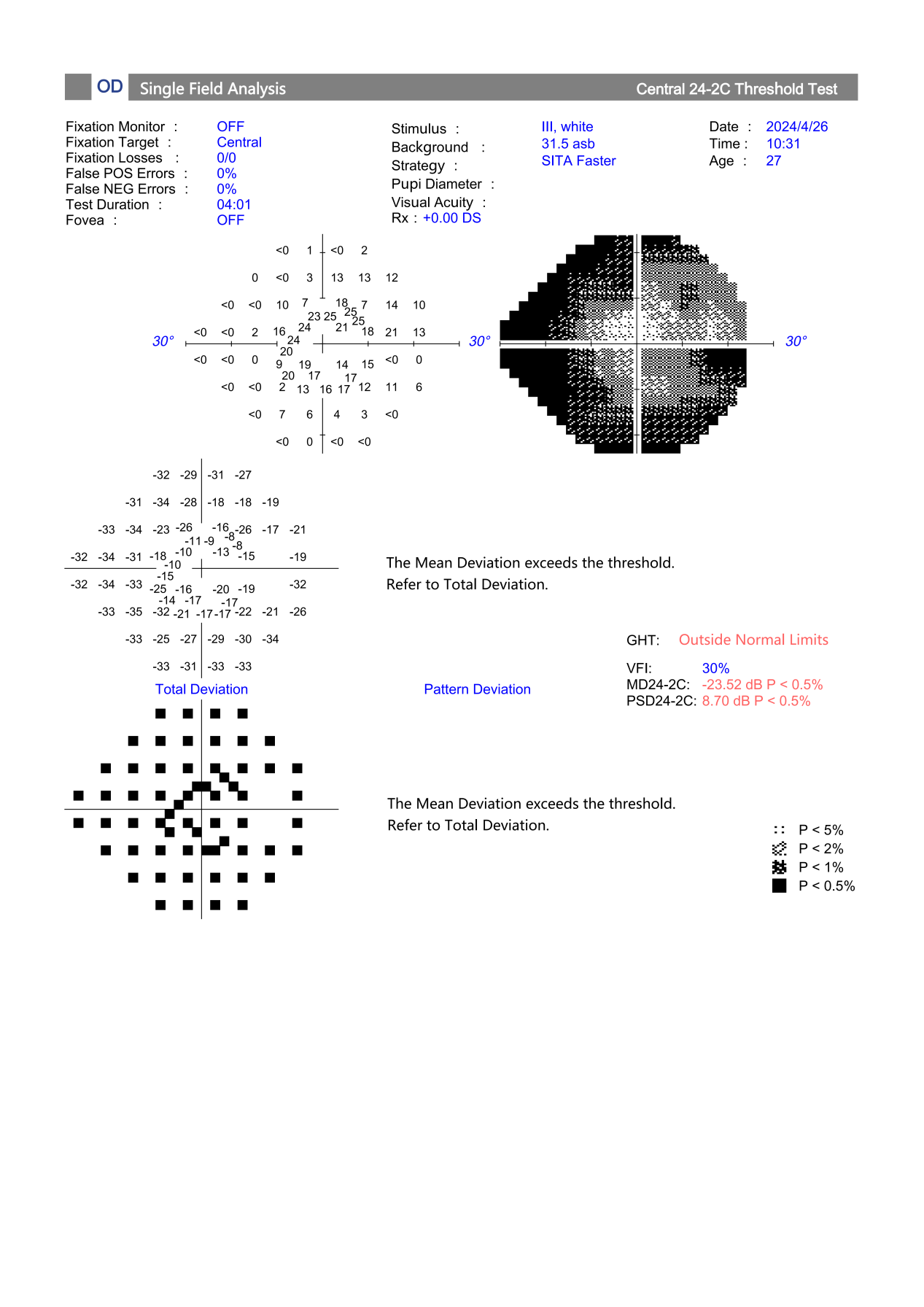
**

**Supplementary Figure. 2** Visual field test of the right eye showing a constricted (tubular) visual field with peripheral loss and central sparing.

**Supplementary Figure. 3** Visual field test of the left eye showing a similar constricted (tubular) visual field pattern.

**
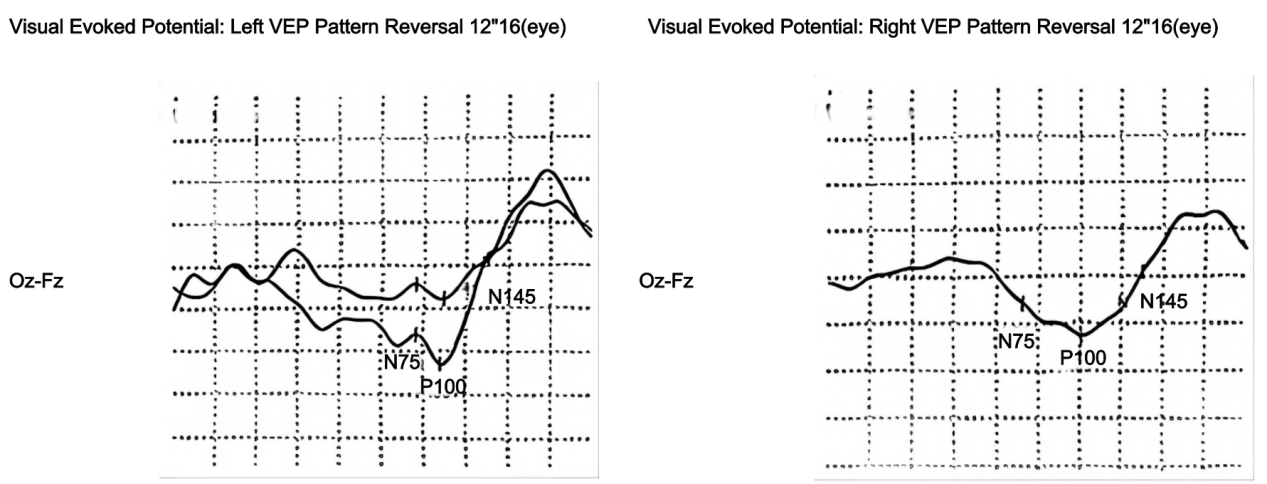
**

**Supplementary Figure. 4 Visual evoked potential waveforms.**

**Supplementary Table. 1 Visual evoked potential parameters**

| Individual Data | | | | | | | | |
| --- | --- | --- | --- | --- | --- | --- | --- | --- |
|  |  |  | Latency | | | Amplitude | | |
|  |  |  | ms | ms | ms | uv | uv | uv |
|  |  |  | Left | Right | Unilateral  Difference | Left | Right | Unilateral  Difference |
| Average | Oz-Fz | N75 | 117 | 92.2 | 24.8 | -3.4 | -2.5 | 0.9 |
|  |  | P100 | 129 | 123 | 6.0 | -6.0 | -6.0 | 0 |
|  |  | N145 | 149 | 150 | 1.00 | 2.3 | 0.67 | 1.63 |
